# (*E*)-3-(4-Chloro­phen­yl)-1-(2,3,4-trichloro­phen­yl)prop-2-en-1-one

**DOI:** 10.1107/S1600536810053213

**Published:** 2011-01-08

**Authors:** Hoong-Kun Fun, Chin Sing Yeap, D. Jagadeesh Prasad, Suresh P. Nayak, K. Laxmana

**Affiliations:** a X-ray Crystallography Unit, School of Physics, Universiti Sains Malaysia, 11800 USM, Penang, Malaysia; b Department of Chemistry, Mangalore University, Mangalore, Karnataka, India

## Abstract

In the title chalcone derivative, C_15_H_8_Cl_4_O, the C=C double bond exists in an *E* configuration and the dihedral angle between the two benzene rings is 48.13 (11)°. In the crystal, mol­ecules are arranged into columns and stacked down the *a* axis featuring possible weak aromatic π–π stacking inter­actions [centroid–centroid separation = 3.888 (2) Å].

## Related literature

For general background to and applications of chalcone derivatives, see: Geiger & Conn (1945 ▶); Misra *et al.* (1971 ▶); Cole & Julian (1954 ▶); Aries (1972 ▶); Levine *et al.* (1979 ▶); Vranasi *et al.* (1996 ▶).
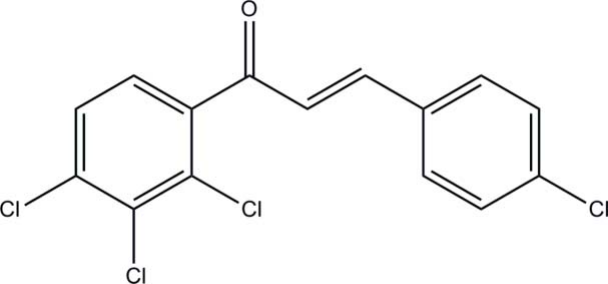


## Experimental

### 

#### Crystal data

C_15_H_8_Cl_4_O
*M* *_r_* = 346.01
Triclinic, 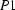

*a* = 3.8879 (2) Å
*b* = 6.7510 (3) Å
*c* = 13.7788 (5) Å
α = 97.620 (2)°
β = 96.177 (2)°
γ = 92.017 (2)°
*V* = 355.93 (3) Å^3^
*Z* = 1
Mo *K*α radiation
μ = 0.82 mm^−1^
*T* = 296 K
0.76 × 0.33 × 0.22 mm

#### Data collection

Bruker SMART APEXII CCD diffractometer
Absorption correction: multi-scan (*SADABS*; Bruker, 2009 ▶) *T*_min_ = 0.574, *T*_max_ = 0.837
7510 measured reflections
3395 independent reflections
3183 reflections with *I* > 2σ(*I*)
*R*_int_ = 0.024

#### Refinement

*R*[*F* ^2^ > 2σ(*F* ^2^)] = 0.030
*wR*(*F* ^2^) = 0.082
*S* = 1.06
3395 reflections
181 parameters
3 restraints
H-atom parameters constrained
Δρ_max_ = 0.33 e Å^−3^
Δρ_min_ = −0.18 e Å^−3^
Absolute structure: Flack (1983 ▶), 1324 Friedel pairs
Flack parameter: −0.02 (5)

### 

Data collection: *APEX2* (Bruker, 2009 ▶); cell refinement: *SAINT* (Bruker, 2009 ▶); data reduction: *SAINT*; program(s) used to solve structure: *SHELXTL* (Sheldrick, 2008 ▶); program(s) used to refine structure: *SHELXTL*; molecular graphics: *SHELXTL*; software used to prepare material for publication: *SHELXTL* and *PLATON* (Spek, 2009 ▶).

## Supplementary Material

Crystal structure: contains datablocks global, I. DOI: 10.1107/S1600536810053213/hb5777sup1.cif

Structure factors: contains datablocks I. DOI: 10.1107/S1600536810053213/hb5777Isup2.hkl

Additional supplementary materials:  crystallographic information; 3D view; checkCIF report

## Figures and Tables

**Fig. 1.**
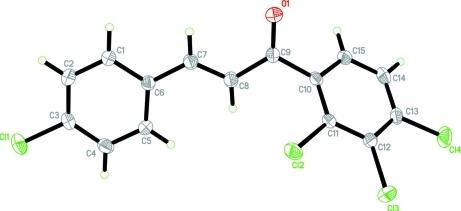
The molecular structure of the title compound with 30% probability ellipsoids for non-H atoms.

**Fig. 2.**
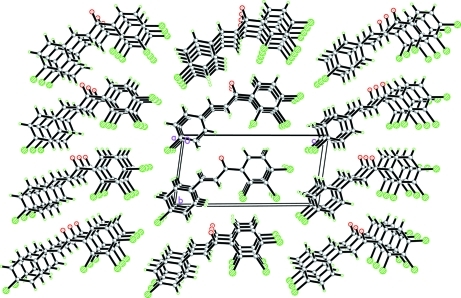
The crystal packing of title compound, viewed down the a axis, showing molecules stacked down a axis.

## supplementary crystallographic information

### Comment

Chalcones are natural biocides (Geiger & Conn, 1945) and also well known as intermediates in the synthesis of heterocyclic compounds which exhibit various biological activities (Misra *et al.*, 1971). The presence of enone functional group in the chalcone molecule confers antibiotic activity upon it (Cole & Julian, 1954). This property is enhanced when substitution is made at the α-(nitro and bromo) and (bromo and hydroxylic-) positions (Aries, 1972). Chalcones are also reported to possess trypanocidal (Levine *et al.*, 1979), anti-inflammatory and anticancer properties (Vranasi *et al.*, 1996).

The C7=C8 double bond of the title compound (I) exists in an *E*-configuration (Fig. 1). The dihedral angle between the two benzene rings being 48.13 (11)°. In the crystal structure, the molecules are arranged into columns and stacked down *a* axis (Fig. 2).

### Experimental

2,3,4-Trichloroacetophenone (10 mmol) was dissolved in ethanol. Sodium hydroxide (5 ml, 30%) solution and 4-chlorobeldehyde (10 mmol) were then added to the resulting solution with continuous stirring. The stirring was continued for 4 h and was allowed to stand overnight. The reaction mass was then poured onto the crushed ice. The resulting solid was separated, filtered and dried. The compound was re-crystallized using ethanol and DMF mixture to yield yellow blocks. M.P.: 162–165 °C

### Refinement

All hydrogen atoms were positioned geometrically [C–H = 0.93 Å] and refined using a riding model, with *U*_iso_(H) = 1.2*U*_eq_(C). A total of 1324 Friedel pairs were use to determine the absolute structure.

### Crystal data

**Table:**

C_15_H_8_Cl_4_O	*Z* = 1
*M**_r_* = 346.01	*F*(000) = 174
Triclinic, *P*1	*D*_x_ = 1.614 Mg m^−^^3^
Hall symbol: P 1	Mo *K*α radiation, λ = 0.71073 Å
*a* = 3.8879 (2) Å	Cell parameters from 5499 reflections
*b* = 6.7510 (3) Å	θ = 3.0–30.0°
*c* = 13.7788 (5) Å	µ = 0.82 mm^−^^1^
α = 97.620 (2)°	*T* = 296 K
β = 96.177 (2)°	Block, yellow
γ = 92.017 (2)°	0.76 × 0.33 × 0.22 mm
*V* = 355.93 (3) Å^3^	

### Data collection

**Table:**

Bruker SMART APEXII CCD diffractometer	3395 independent reflections
Radiation source: fine-focus sealed tube	3183 reflections with *I* > 2σ(*I*)
graphite	*R*_int_ = 0.024
φ and ω scans	θ_max_ = 30.0°, θ_min_ = 1.5°
Absorption correction: multi-scan (*SADABS*; Bruker, 2009)	*h* = −5→5
*T*_min_ = 0.574, *T*_max_ = 0.837	*k* = −8→9
7510 measured reflections	*l* = −19→19

### Refinement

**Table:**

Refinement on *F*^2^	Secondary atom site location: difference Fourier map
Least-squares matrix: full	Hydrogen site location: inferred from neighbouring sites
*R*[*F*^2^ > 2σ(*F*^2^)] = 0.030	H-atom parameters constrained
*wR*(*F*^2^) = 0.082	*w* = 1/[σ^2^(*F*_o_^2^) + (0.0435*P*)^2^ + 0.0513*P*] where *P* = (*F*_o_^2^ + 2*F*_c_^2^)/3
*S* = 1.06	(Δ/σ)_max_ < 0.001
3395 reflections	Δρ_max_ = 0.33 e Å^−^^3^
181 parameters	Δρ_min_ = −0.18 e Å^−^^3^
3 restraints	Absolute structure: Flack (1983), 1324 Friedel pairs
Primary atom site location: structure-invariant direct methods	Flack parameter: −0.02 (5)

### Special details

**Table:**

Geometry. All e.s.d.'s (except the e.s.d. in the dihedral angle between two l.s. planes) are estimated using the full covariance matrix. The cell e.s.d.'s are taken into account individually in the estimation of e.s.d.'s in distances, angles and torsion angles; correlations between e.s.d.'s in cell parameters are only used when they are defined by crystal symmetry. An approximate (isotropic) treatment of cell e.s.d.'s is used for estimating e.s.d.'s involving l.s. planes.
Refinement. Refinement of *F*^2^ against ALL reflections. The weighted *R*-factor *wR* and goodness of fit *S* are based on *F*^2^, conventional *R*-factors *R* are based on *F*, with *F* set to zero for negative *F*^2^. The threshold expression of *F*^2^ > σ(*F*^2^) is used only for calculating *R*-factors(gt) *etc*. and is not relevant to the choice of reflections for refinement. *R*-factors based on *F*^2^ are statistically about twice as large as those based on *F*, and *R*- factors based on ALL data will be even larger.

### Fractional atomic coordinates and isotropic or equivalent isotropic displacement parameters (Å^2^)

**Table:**

	*x*	*y*	*z*	*U*_iso_*/*U*_eq_	
Cl1	0.18842 (18)	1.21292 (10)	−0.08423 (5)	0.05987 (19)	
Cl2	1.09977 (14)	0.85687 (8)	0.46292 (4)	0.04498 (14)	
Cl3	0.99789 (19)	0.85943 (10)	0.68403 (5)	0.05664 (17)	
Cl4	0.67317 (19)	0.47391 (11)	0.75284 (5)	0.0645 (2)	
O1	0.9846 (5)	0.3188 (3)	0.28530 (12)	0.0512 (4)	
C1	0.5993 (6)	0.7313 (4)	0.00979 (16)	0.0407 (5)	
H1A	0.6920	0.6148	−0.0180	0.049*	
C2	0.4793 (7)	0.8687 (4)	−0.05019 (16)	0.0445 (5)	
H2A	0.4894	0.8451	−0.1178	0.053*	
C3	0.3445 (6)	1.0413 (4)	−0.00805 (17)	0.0399 (5)	
C4	0.3247 (6)	1.0802 (4)	0.09119 (18)	0.0428 (5)	
H4A	0.2311	1.1973	0.1179	0.051*	
C5	0.4465 (6)	0.9424 (3)	0.15142 (15)	0.0386 (5)	
H5A	0.4366	0.9685	0.2190	0.046*	
C6	0.5836 (5)	0.7650 (3)	0.11160 (14)	0.0336 (4)	
C7	0.7206 (6)	0.6183 (3)	0.17296 (15)	0.0369 (4)	
H7A	0.8342	0.5133	0.1418	0.044*	
C8	0.6995 (6)	0.6198 (3)	0.26870 (15)	0.0372 (4)	
H8A	0.5872	0.7226	0.3021	0.045*	
C9	0.8480 (6)	0.4641 (3)	0.32376 (14)	0.0348 (4)	
C10	0.8101 (5)	0.4806 (3)	0.43238 (14)	0.0319 (4)	
C11	0.9116 (5)	0.6485 (3)	0.50096 (15)	0.0309 (4)	
C12	0.8730 (6)	0.6515 (3)	0.60100 (16)	0.0357 (4)	
C13	0.7287 (6)	0.4796 (4)	0.63117 (16)	0.0384 (5)	
C14	0.6308 (6)	0.3128 (3)	0.56447 (18)	0.0432 (5)	
H14A	0.5364	0.1997	0.5856	0.052*	
C15	0.6713 (6)	0.3117 (3)	0.46607 (16)	0.0384 (4)	
H15A	0.6054	0.1972	0.4216	0.046*	

### Atomic displacement parameters (Å^2^)

**Table:**

	*U*^11^	*U*^22^	*U*^33^	*U*^12^	*U*^13^	*U*^23^
Cl1	0.0665 (4)	0.0563 (4)	0.0629 (4)	0.0105 (3)	0.0037 (3)	0.0313 (3)
Cl2	0.0504 (3)	0.0319 (2)	0.0539 (3)	−0.0034 (2)	0.0081 (2)	0.0100 (2)
Cl3	0.0731 (4)	0.0484 (3)	0.0425 (3)	0.0058 (3)	−0.0047 (3)	−0.0069 (2)
Cl4	0.0849 (5)	0.0797 (5)	0.0369 (3)	0.0232 (4)	0.0178 (3)	0.0231 (3)
O1	0.0688 (12)	0.0460 (9)	0.0405 (8)	0.0188 (9)	0.0118 (8)	0.0036 (7)
C1	0.0496 (13)	0.0391 (11)	0.0343 (10)	0.0064 (10)	0.0069 (9)	0.0059 (8)
C2	0.0545 (13)	0.0489 (12)	0.0309 (9)	0.0041 (11)	0.0054 (9)	0.0072 (9)
C3	0.0393 (11)	0.0390 (11)	0.0440 (11)	0.0012 (9)	0.0028 (9)	0.0170 (9)
C4	0.0469 (12)	0.0331 (10)	0.0504 (12)	0.0074 (9)	0.0096 (10)	0.0078 (9)
C5	0.0468 (12)	0.0374 (11)	0.0314 (9)	0.0025 (9)	0.0056 (9)	0.0027 (8)
C6	0.0355 (9)	0.0342 (9)	0.0323 (9)	0.0014 (8)	0.0054 (8)	0.0074 (7)
C7	0.0376 (10)	0.0379 (11)	0.0367 (10)	0.0050 (9)	0.0061 (8)	0.0085 (8)
C8	0.0388 (11)	0.0399 (11)	0.0348 (10)	0.0067 (9)	0.0057 (8)	0.0095 (8)
C9	0.0379 (10)	0.0357 (10)	0.0317 (9)	0.0035 (8)	0.0044 (8)	0.0073 (7)
C10	0.0347 (10)	0.0293 (9)	0.0334 (9)	0.0074 (8)	0.0047 (8)	0.0086 (7)
C11	0.0307 (9)	0.0281 (9)	0.0348 (9)	0.0051 (7)	0.0024 (7)	0.0079 (7)
C12	0.0372 (10)	0.0364 (10)	0.0332 (9)	0.0105 (8)	−0.0003 (8)	0.0045 (7)
C13	0.0409 (11)	0.0460 (12)	0.0318 (9)	0.0127 (9)	0.0046 (8)	0.0146 (8)
C14	0.0462 (12)	0.0397 (12)	0.0483 (12)	0.0046 (10)	0.0075 (10)	0.0203 (10)
C15	0.0469 (12)	0.0286 (10)	0.0388 (10)	−0.0008 (8)	0.0000 (9)	0.0063 (8)

### Geometric parameters (Å, °)

**Table:**

Cl1—C3	1.745 (2)	C6—C7	1.463 (3)
Cl2—C11	1.730 (2)	C7—C8	1.329 (3)
Cl3—C12	1.709 (2)	C7—H7A	0.9300
Cl4—C13	1.718 (2)	C8—C9	1.474 (3)
O1—C9	1.218 (3)	C8—H8A	0.9300
C1—C2	1.383 (3)	C9—C10	1.509 (3)
C1—C6	1.399 (3)	C10—C11	1.392 (3)
C1—H1A	0.9300	C10—C15	1.399 (3)
C2—C3	1.378 (3)	C11—C12	1.400 (3)
C2—H2A	0.9300	C12—C13	1.403 (3)
C3—C4	1.369 (3)	C13—C14	1.370 (4)
C4—C5	1.389 (3)	C14—C15	1.381 (3)
C4—H4A	0.9300	C14—H14A	0.9300
C5—C6	1.398 (3)	C15—H15A	0.9300
C5—H5A	0.9300		
			
C2—C1—C6	121.0 (2)	C9—C8—H8A	118.8
C2—C1—H1A	119.5	O1—C9—C8	123.42 (18)
C6—C1—H1A	119.5	O1—C9—C10	118.57 (18)
C3—C2—C1	118.9 (2)	C8—C9—C10	117.92 (17)
C3—C2—H2A	120.6	C11—C10—C15	118.23 (18)
C1—C2—H2A	120.6	C11—C10—C9	124.79 (18)
C4—C3—C2	122.0 (2)	C15—C10—C9	116.95 (19)
C4—C3—Cl1	119.28 (18)	C10—C11—C12	121.51 (18)
C2—C3—Cl1	118.73 (17)	C10—C11—Cl2	119.60 (14)
C3—C4—C5	119.1 (2)	C12—C11—Cl2	118.87 (16)
C3—C4—H4A	120.5	C11—C12—C13	118.2 (2)
C5—C4—H4A	120.5	C11—C12—Cl3	120.83 (17)
C4—C5—C6	120.77 (19)	C13—C12—Cl3	120.94 (16)
C4—C5—H5A	119.6	C14—C13—C12	120.76 (19)
C6—C5—H5A	119.6	C14—C13—Cl4	118.73 (17)
C5—C6—C1	118.25 (19)	C12—C13—Cl4	120.51 (18)
C5—C6—C7	122.28 (18)	C13—C14—C15	120.4 (2)
C1—C6—C7	119.43 (18)	C13—C14—H14A	119.8
C8—C7—C6	126.72 (19)	C15—C14—H14A	119.8
C8—C7—H7A	116.6	C14—C15—C10	120.9 (2)
C6—C7—H7A	116.6	C14—C15—H15A	119.6
C7—C8—C9	122.32 (19)	C10—C15—H15A	119.6
C7—C8—H8A	118.8		
			
C6—C1—C2—C3	0.4 (4)	C8—C9—C10—C15	127.9 (2)
C1—C2—C3—C4	−0.3 (4)	C15—C10—C11—C12	−0.9 (3)
C1—C2—C3—Cl1	−179.2 (2)	C9—C10—C11—C12	−178.96 (19)
C2—C3—C4—C5	0.5 (4)	C15—C10—C11—Cl2	177.53 (17)
Cl1—C3—C4—C5	179.44 (19)	C9—C10—C11—Cl2	−0.6 (3)
C3—C4—C5—C6	−0.8 (4)	C10—C11—C12—C13	0.1 (3)
C4—C5—C6—C1	0.9 (3)	Cl2—C11—C12—C13	−178.29 (16)
C4—C5—C6—C7	178.7 (2)	C10—C11—C12—Cl3	−179.78 (17)
C2—C1—C6—C5	−0.7 (3)	Cl2—C11—C12—Cl3	1.8 (2)
C2—C1—C6—C7	−178.6 (2)	C11—C12—C13—C14	0.5 (3)
C5—C6—C7—C8	8.2 (4)	Cl3—C12—C13—C14	−179.62 (19)
C1—C6—C7—C8	−174.0 (2)	C11—C12—C13—Cl4	−179.80 (16)
C6—C7—C8—C9	−179.9 (2)	Cl3—C12—C13—Cl4	0.1 (3)
C7—C8—C9—O1	−3.7 (4)	C12—C13—C14—C15	−0.3 (3)
C7—C8—C9—C10	179.8 (2)	Cl4—C13—C14—C15	179.98 (19)
O1—C9—C10—C11	129.4 (2)	C13—C14—C15—C10	−0.5 (4)
C8—C9—C10—C11	−53.9 (3)	C11—C10—C15—C14	1.1 (3)
O1—C9—C10—C15	−48.7 (3)	C9—C10—C15—C14	179.3 (2)
